# Advancing Dermatologic Equity for Individuals With Autism Through Awareness and Structural Reform

**DOI:** 10.7759/cureus.83829

**Published:** 2025-05-10

**Authors:** Radhika Misra, Renee Chang, Olumayowa T Adebiyi, Maria Orbe, Donna Pham, Kaitlyn Miner, Kelly M Frasier

**Affiliations:** 1 College of Osteopathic Medicine, Des Moines University, West Des Moines, USA; 2 Department of Dermatology, Touro College of Osteopathic Medicine, Henderson, USA; 3 Department of Internal Medicine, St. John's Riverside Hospital, Yonkers, USA; 4 Department of Dermatology, University of Missouri School of Medicine, Columbia, USA; 5 Department of Dermatology, University of California Riverside School of Medicine, Riverside, USA; 6 College of Medicine, Kansas City University, Kansas City, USA; 7 Department of Dermatology, Northwell Health, New Hyde Park, USA

**Keywords:** autism spectrum disease, autism spectrum disorder (asd), barriers to care, clinical dermatology, neurodevelopmental disorders

## Abstract

Autism spectrum disorder (ASD), a neurodevelopmental condition affecting communication, social interaction, and sensory processing, has seen a marked increase in prevalence over the past two decades. Dermatologists are increasingly encountering patients with ASD, many of whom face compounded challenges due to mobility issues, such as reliance on wheelchairs, as well as sensory hypersensitivities and communication barriers. Despite the growing demand for specialized care, advocacy for accessible dermatologic health for individuals with ASD remains insufficient. This literature review highlights the challenges faced by this population, including higher susceptibility to certain dermatologic conditions and the significant barriers to effective clinical evaluation and management. By identifying key gaps in care, this review advocates for the implementation of sensory-aware practices, such as adapting clinical environments, employing tailored communication strategies, and designing individualized treatment plans. Furthermore, it emphasizes the critical role of dermatologists, researchers, and policymakers in championing systemic changes that address these barriers and promote equitable access to care. Through a commitment to advocacy and innovation, dermatology can better meet the needs of individuals with ASD and contribute to advancing skin health equity on a global scale.

## Introduction and background

Advocacy in dermatology involves addressing systemic barriers to equitable care and improving outcomes for diverse patient populations. The American Medical Association (AMA) defines advocacy as a commitment to pursuing "social, economic, educational, and political changes that ameliorate suffering and contribute to human well-being" [1]. While the American Academy of Dermatology (AAD) endorses advocacy priorities like skin cancer prevention and expanding treatment access, comprehensive health advocacy must also tackle the systemic drivers of health disparities. For individuals with autism spectrum disorder (ASD), advocacy encompasses understanding their unique challenges, including communication barriers, sensory sensitivities, and mobility issues, and ensuring dermatologic care meets their distinct needs. Resources such as AutismSpeaks.org provide valuable insights into strategies for clinicians and families, emphasizing the need to integrate biological, social, and behavioral considerations into care for patients with ASD.

The growing prevalence of ASD underscores its critical implications for dermatologic care. In 2002, the prevalence of ASD in the United States was estimated to affect one in 150 children, and, by 2010, this had increased to one in 66 children. By 2025, the prevalence has risen further to one in 36 children, as estimated by the Autism and Developmental Disabilities Monitoring (ADDM) Network of the Centers for Disease Control and Prevention (CDC) [2]. This represents a staggering 316.7% increase in prevalence from 2002 to 2025, reflecting substantial progress in diagnostic awareness and reporting. Despite this progress, gaps remain, particularly in equitable access to care. ASD affects all racial, ethnic, and socioeconomic groups, with a notable increase in diagnoses among non-White children and girls [2,3]. These trends highlight an urgent need for advocacy to ensure equitable access to diagnostic, treatment, and support services for all children with ASD. Addressing this growing population's needs requires dermatologists to actively mitigate barriers like sensory sensitivities to touch, aversion to specific textures, and difficulties in articulating symptoms.

Advocacy must operate at individual, community, and policy levels to address these disparities in dermatologic care for patients with ASD. Over the past two decades, the global dermatologic community has achieved remarkable progress in research, programming, and funding to address the burden of skin conditions [4]. Skin diseases are now recognized as the fourth leading cause of non-fatal disease burden worldwide, and, similarly, ASD ranks among the top 10 causes of non-fatal health burden for young people under 20 years of age, highlighting the need for greater attention to the intersection of these conditions [5,6]. As dermatology expands its focus on health equity, advocacy efforts must align with the principles of inclusive care to ensure that patients with ASD receive comprehensive, equitable treatment. The purpose of this review is to raise awareness of the unique challenges faced by individuals with ASD in accessing equitable dermatologic care and to address the barriers that hinder the delivery of inclusive, patient-centered solutions.

## Review

Common skin-related manifestations in ASD

Eczematous dermatitis, including atopic dermatitis (AD), is among the most common skin disorders in individuals with ASD, highlighting an important intersection between dermatologic and neurodevelopmental health. Increasing evidence suggests that skin abnormalities, such as AD and eczema, may contribute to an elevated risk of neurodevelopmental disorders, with effects potentially beginning in early childhood [7-9]. This connection is supported by immunological mechanisms, particularly elevated levels of pro-inflammatory cytokines like interleukin 17 (IL-17), which can influence neurodevelopment during critical periods [10]. Epidemiological studies reveal a consistently higher prevalence of AD in children with ASD compared to controls, with rates ranging from 28.4% in ASD populations to 15.4% in non-ASD populations and over 52% of individuals with ASD presenting with allergic manifestations [11,12]. The association appears to be influenced by AD severity, as severe cases correlate with increased ASD risk, while mild or moderate cases show no significant association [13,14]. Although some studies have reported conflicting findings, likely due to variations in study design and population characteristics, the cumulative evidence demonstrates a complex interplay between systemic inflammation, immune dysregulation, and neurodevelopment in ASD, emphasizing the need for further research to explore their clinical implications.

Psoriasis, a chronic inflammatory skin condition, has been found to be more common in children with ASD than in those without it, especially in those with a positive family history, particularly maternal psoriasis during pregnancy [13]. Studies show that children with ASD are twice as likely to have psoriasis compared to children without ASD (0.34% vs. 0.15%) [15]. This connection may be driven by inflammation involving the cytokine IL-17A, which is elevated in both psoriasis and ASD. IL-17A is thought to influence brain development by inducing neuroinflammation, as demonstrated in mice where injecting IL-17A into the fetal brain led to ASD-like behaviors [16]. Blocking IL-17A, on the other hand, has been shown to improve these symptoms in animal models. In humans, treatments for psoriasis that inhibit IL-17A have also been observed to alleviate psychological symptoms in individuals with ASD [17]. These findings emphasize the need for advocacy in dermatology to address the unique challenges faced by individuals with ASD, including the recognition of psoriasis as a comorbidity, improving access to treatments targeting shared inflammatory pathways, and promoting multidisciplinary care to enhance patient outcomes.

Hyperpigmented skin conditions, including pigmentary mosaicism and café-au-lait macules (CALMs), have been observed at higher rates in individuals with ASD, suggesting potential shared pathophysiological mechanisms. Studies indicate that pigmentary mosaicism of the hyperpigmented type is significantly associated with ASD (OR = 2.76; P = 0.02), as are CALMs, with a less pronounced but still notable trend for atypical CALMs [7]. This association may stem from underlying genetic or epigenetic disruptions that concurrently affect neurodevelopment and melanocyte biology. For example, mutations in genes such as neurofibromatosis type 1 (NF1) and SH3 and multiple ankyrin repeat domains 3 (SHANK3), which are implicated in CALMs and ASD, respectively, underscore potential overlaps in developmental pathways. Hyperpigmentation along the lines of Blaschko, observed in some cases of ASD, further suggests a role for somatic mosaicism in this comorbidity [18]. These findings further emphasize the need for dermatologic advocacy to address challenges in recognition, access to care, and treatment for individuals with ASD (Table 1).

**Table 1:** Comparison of skin conditions prevalent among individuals with ASD. CALMs: café-au-lait macules; IL-17: interleukin 17; ASD: autism spectrum disorder; NF1: neurofibromatosis type 1; SHANK3: SH3 and multiple ankyrin repeat domains 3

Skin condition	Prevalence in ASD	Key findings	Mechanisms	Clinical implications
Atopic dermatitis/eczema	28.4% in ASD vs. 15.4% in non-ASD; >52% with allergic features [11,12]	Strong association with ASD, especially in severe cases	Elevated IL-17 and other pro-inflammatory cytokines affecting neurodevelopment	Need for early dermatologic and neurologic screening; importance of managing inflammation
Psoriasis	0.34% in ASD vs. 0.15% in non-ASD [15]	Increased prevalence in ASD, especially with maternal history of psoriasis	Elevated IL-17A induces neuroinflammation; blocking IL-17A improves ASD-like symptoms in mice	Advocate for recognizing psoriasis as a comorbidity in ASD; access to IL-17A-targeting therapies
Hyperpigmented conditions (e.g., CALMs, pigmentary mosaicism)	OR = 2.76 for pigmentary mosaicism; increased rate of CALMs [7]	Higher occurrence of pigmentary anomalies in ASD; overlaps with genetic syndromes	Genetic/epigenetic disruptions in NF1 and SHANK3; somatic mosaicism	Importance of dermatologic exams in ASD; consider genetic evaluation for patients with multiple CALMs

Challenges in dermatologic advocacy for individuals with ASD 

Structural inequities in healthcare distribution extend beyond rural and underserved areas, creating significant barriers for individuals with ASD across various geographic and demographic contexts. For minoritized communities, these inequities amplify challenges in accessing specialized dermatologic care, resulting in a critical shortage of services tailored to the needs of neurodiverse patients and further widening existing health disparities [19]. Research highlights that minoritized and Hispanic individuals with ASD are disproportionately impacted, with significantly less access to healthcare compared to their White, American Indian, and Alaska Native counterparts [19]. While telemedicine holds the potential for bridging gaps in care, it introduces other challenges for individuals with ASD, including discomfort with video calls, lack of accommodations designed for neurodiverse users, and difficulties in obtaining high-quality images for accurate diagnosis [20]. These challenges necessitate comprehensive and innovative solutions that address both telehealth and in-person care to ensure accessibility and equity.

**Figure 1:**
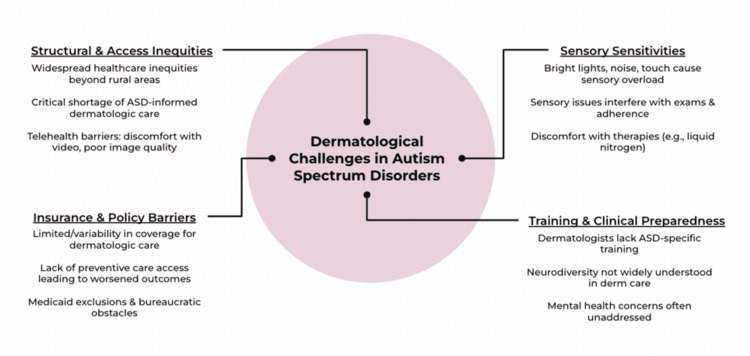
Four key domains characterize the dermatologic care challenges faced by individuals with ASD. Structural and access inequities include a shortage of ASD-informed dermatologists, limited telehealth usability due to sensory sensitivities, and disparities that extend beyond rural settings. Insurance and policy barriers involve inconsistent coverage, poor access to preventive care, and exclusions within Medicaid and related systems. Sensory sensitivities, such as heightened responses to light, sound, and touch, can disrupt examinations and impair treatment adherence, particularly with aversive procedures. Training and clinical preparedness remains limited, with insufficient ASD-specific education, minimal integration of neurodiversity principles, and frequent oversight of mental health comorbidities. Together, these domains emphasize the need for a neurodiversity-informed dermatologic care model grounded in sensory-adaptive strategies, clinician training, and systemic reform. ASD: autism spectrum disorder

Sensory hypersensitivities in individuals with ASD can interfere with dermatologic evaluations, requiring sensory-friendly adaptions and ASD-informed care models to improve clinical experiences, treatment adherence, and psychosocial outcomes. Bright lights, loud noises, and tactile stimuli may trigger sensory overload and exacerbate anxiety, leading to agitation during or avoidance of clinic visits [21]. Heightened sensitivity to touch can make routine medical procedures particularly challenging, emphasizing the need for sensory-friendly adaptations to enhance physician-patient interactions [22]. Moreover, sensory difficulties may also affect treatment adherence, further highlighting the importance of tailored approaches in care delivery [22]. Discomfort with the textures or temperatures of therapies, such as liquid nitrogen or topical agents, may necessitate personalized treatment plans and regular follow-up appointments to promote adherence. Effectively accommodating sensory sensitivities in individuals with ASD requires a collaborative approach, integrating dermatologic care with behavioral health strategies. However, there is limited integration between dermatology and behavioral or developmental health professionals, despite evidence that a multidisciplinary approach, including dermatologists, psychiatrists, and psychologists, is required to address this complex interplay between dermatoses and mental health [23,24]. Multidisciplinary management strategies can improve patient outcomes by addressing sensory barriers and psychosocial needs. Dermatologic conditions often have significant psychosocial implications, including heightened social anxiety and self-isolation, which are compounded by the stigma surrounding an ASD diagnosis [25,26]. While developing informed care models can enhance clinical encounters and treatment adherence, access to inclusive services and multidisciplinary care remains a significant challenge for this population (Figure 1). 

These challenges are compounded by policy and structural barriers, with state-level variability in insurance mandates creating significant obstacles to accessing dermatologic care for individuals with ASD. Limited insurance coverage for dermatologic treatments poses challenges for individuals with ASD, contributing to disparities in healthcare access. Despite the need for ongoing medical support, particularly for conditions related to ASD, insurance policies often do not adequately cover dermatologic care, creating financial and administrative obstacles for patients and caregivers [27]. Individuals with ASD frequently experience sensory sensitivities and dermatologic conditions such as eczema, which may require specialized care. However, restrictive insurance policies limit access to timely treatment, potentially leading to prolonged discomfort and complications. 

Insurance coverage for preventive dermatologic care remains limited, particularly for routine skin monitoring and early interventions, which can lead to worsened outcomes for individuals with ASD. Exclusions in coverage are further compounded by barriers within Medicaid and state-based programs, resulting in delayed access for many autistic individuals who may struggle to advocate for their own needs [28]. Such restrictions can lead to missed opportunities for the early detection of inflammatory or potentially serious conditions, as well as heightened anxiety for patients with sensory processing differences. Research suggests that when preventive services are not prioritized, individuals with ASD risk more frequent use of urgent or emergent care, potentially increasing healthcare costs and exacerbating stress [29]. Efforts to address these gaps include advocating for expanded Medicaid reimbursement, improving insurance coverage structures, and enhancing provider training to support timely, tailored interventions [30]. These measures may align resources more effectively, reduce the financial burden on families, and foster improved long-term health outcomes. Promoting equitable access to preventive services could help dismantle systemic barriers, offering individuals with ASD a more integrated and supportive healthcare experience.

In addition to financial barriers, the absence of ASD-specific training among dermatologists may further limit access to appropriate care. There are no standardized requirements mandating dermatology professionals to receive training on the unique needs of autistic individuals, which may contribute to miscommunication, inadequate treatment approaches, and patient distress [31]. The neurodiversity movement has emphasized the importance of shifting towards evidence-based, patient-centered care rather than behavior-modifying interventions. However, these approaches are not widely integrated into dermatologic care, leaving many providers unprepared to address ASD-related concerns effectively. Studies suggest that individuals with ASD face higher rates of mental health challenges, which may be exacerbated by financial and systemic barriers to care [32]. Expanding insurance coverage and integrating ASD-specific training into dermatology education may help address these challenges and improve access to appropriate care.

Advocacy in action: strategies and initiatives

Advocacy directed at addressing the barriers to accessing inclusive dermatologic care for individuals with ASD begins with initiatives that reduce the stigma associated with an ASD diagnosis through public health campaigns and community outreach programs. Several organizations currently exist that provide resources for the autistic community, including support groups, employment opportunities, and providing communities with education on supporting individuals with ASD. SPARK for Autism, for example, is a research-based organization that aims to improve the quality of life of those with autism by providing individuals and their caregivers with evidence-based information regarding autism [33]. Through community advocacy efforts, SPARK for Autism is reducing the stigma surrounding an autism diagnosis by increasing social awareness and encouraging supportive communities. While destigmatization is an important first step in advocacy for individuals with ASD, gaps in advocacy and research still exist, particularly at the intersection of dermatologic conditions and autism. Partnering professional health groups with a well-established research advocate such as SPARK for Autism may bridge this gap by integrating input from experts in multiple disciplines to create specific initiatives tailored to address skin health in individuals with ASD. Having AAD implement campaigns to address the dermatologic comorbidities of ASD, similar to those they hold for conditions like rosacea or acne, holds significant potential to enhance education and awareness, ultimately addressing the existing gaps in care. Through partnership with research-based advocacy groups like SPARK for Autism, ASD-inclusive care models can be developed that address the sensory processing barriers associated with dermatologic evaluations, procedures, and treatments. By working together, organizations like the AAD and SPARK for Autism can decrease stigma and improve dermatologic care in individuals with ASD by spreading awareness, educating communities and caregivers, and creating initiatives that address barriers to treatment.

Extending beyond professional organizations, advocacy through community outreach programs plays a vital role in addressing the unmet dermatologic needs of the autism community by providing accessible education at the local level. Community outreach programs such as PArticipation in Rural Settings to Engage in Communities (PARSEC), which is an initiative designed to support families of young adults with autism in rural areas by enhancing their community participation, can have a greater impact on the dermatologic health of ASD individuals [34]. By using an outreach program such as PARSEC, adaptation of dermatologic health can greatly improve by creating sensory-friendly materials explaining skin health and procedures, preparing them for appointments, or even partnering with dermatologists to advocate for autism-specific needs. In addition, educating patients as well as caregivers is an additional tool that would improve their quality of care. A scoping review demonstrated that medical student-led educational interventions within the local community can improve post-interventional health education and perspectives [35]. While this study focused on education pertaining to skin cancer prevention, it highlights a broader opportunity to extend similar educational initiatives to autistic communities and support groups for caregivers. Educating caregivers and individuals with autism about dermatologic comorbidities and providing resources for accessible care can decrease the time to diagnosis and treatment, ultimately improving outcomes in this population. Collaborating with organizations such as the American Autism Association can improve caregiver education through their emphasis on advocacy, educational workshops, and therapeutic recreational programs [36]. Education about stigmatized diagnoses plays a powerful role in destigmatizing; using educational programming to raise awareness about the dermatologic comorbidities associated with autism may encourage the creation of more inclusive therapeutic interventions.

Addressing the barriers faced by individuals with ASD not only requires community outreach and education but also necessitates policy advocacy focused on improving insurance coverage and sensory-friendly infrastructure in healthcare settings. Those with ASD already face the burden of excessive healthcare costs, exceeding an additional $3400 in annual expenses compared to the non-ASD population [37]. There have been mandates implemented under the Mental Health Parity and Addiction Equity Act to reduce the financial burden on individuals with ASD, such as requiring the copay for autism service providers to be no higher than normal medical care costs [38]; however, ASD-specific dermatologic care interventions are lacking. Regular healthcare appointments are an essential part of managing the numerous comorbidities associated with ASD. Healthcare settings, however, can be overly stimulating and can cause distress and agitation in ASD patients [39]. This underscores the importance of sensory-friendly infrastructure in healthcare settings to accommodate their needs and ensure that their care is less distressing and more accessible. To create an ASD-friendly environment, some healthcare settings, like emergency rooms, have introduced sensory equipment such as noise-canceling headphones, ambient sounds, and other tools, which have proven effective in improving patient attitudes and willingness to cooperate [40]. Creating sensory-adapted environments for ASD patients within dermatologic care is essential, given the necessity of performing skin examinations and potential procedures. In addition to addressing sensory barriers during appointments, communication challenges in scheduling initial appointments have also been a proven barrier for individuals with ASD in accessing healthcare. In a cross-sectional study of over 500 autistic adults and over 100 non-autistic adults in 2022, researchers demonstrated that 72% of autistic adults experience difficulty getting an appointment with a general practitioner, including 53% reporting challenges in effectively communicating with their general practitioner regarding the availability of an appointment [41]. This highlights the role of patient advocates in healthcare settings, such as social workers and caregivers, who play an essential role in addressing communication barriers between healthcare systems and individuals with ASD. By prioritizing sensory-friendly adaptations, targeting policy changes, and improving accessibility in healthcare settings, healthcare systems can address the barriers faced by the autism community in a dermatologic setting.

Case studies in advocacy 

There have been many successful ASD initiatives implemented in the clinical setting. Addressing sensory sensitivities in individuals with ASD has led to the development of several innovative multimedia interventions in medical practice, especially in rehabilitation and therapy. Among these, the integration of virtual reality (VR) technologies has shown promising potential for manipulating sensory, motor, interpersonal, and cognitive processes in healthcare settings [42]. VR offers immersive environments that can be customized to meet the specific sensory needs of individuals with ASD, providing controlled exposure to stimuli in a safe and supportive setting. This technology enables clinicians to design tailored interventions that enhance therapeutic engagement and outcomes. For example, Frolli et al. demonstrated that the use of VR improved the acquisition times for recognizing primary emotions in individuals with ASD compared to those who did not engage with VR [43]. Similarly, Maskey et al. found that VR interventions effectively reduced or treated specific phobias in patients with ASD through graduated exposure, a method that allows for incremental desensitization to feared stimuli in a virtual environment [44]. These immersive VR environments not only address sensory sensitivities but also aid in the development of critical behavioral and social skills [44]. In dermatology, VR can be used as distraction therapy to help ASD patients with psoriasis or eczema manage stress levels during treatment sessions. In addition, VR can also help ASD patients better visualize a model of their skin and what to expect for surgery or treatments. VR-based interventions hold significant promise for improving the quality of life and clinical outcomes for individuals with ASD by addressing their unique sensory and behavioral needs.

Policy advocacy plays a pivotal role in ensuring equitable access to healthcare services for individuals with disabilities. Choi et al. examine the role of policy advocacy in the implementation of autism insurance mandates across the United States [45]. While most states provide coverage for provider-recommended ASD services, limitations exist in several areas. These include exclusions for medical equipment, coverage caps at ages 18-21, and annual financial limits such as $36,000 without restrictions on service hours or visits. Providers of applied behavior analysis (ABA) are typically required to meet certification standards, but dermatologic care is not explicitly included in these mandates [45]. This highlights the necessity for ongoing research to evaluate the impact of these mandates on access to care, service utilization, and clinical outcomes for the ASD population. The findings suggest that stakeholder engagement and understanding patient-centered outcomes are crucial for guiding future policy advocacy and public health initiatives. Moreover, the Americans with Disabilities Act (ADA) prohibits discrimination against individuals with disabilities and ensures equal access to medical services [46]. However, this does not take into consideration the sensory and communication barriers faced by the autism community seeking subspecialty care such as dermatology. Given the lack of specific mandates for dermatologic care in ASD, it is crucial for healthcare providers to be aware of the potential dermatological needs of individuals with autism. Specific mandates that integrate dermatologic care into the broader framework of disability-focused healthcare policies are needed. 

There have been many community-driven programs that have demonstrated efficacy in enhancing health literacy and access for ASD populations. The Extension for Community Healthcare Outcomes (ECHO) program allows ASD experts to provide guided practice to professionals in local communities on evidence-based care for children with ASD and their families [47]. The ECHO model educates clinicians on screening, diagnosing, and managing ASD. This model's adaptability to local, regional, and cultural contexts allows for global application. Additionally, technological innovations, such as mobile applications, have been developed to identify autism in areas of the world with limited access to specialists. Many autistic patients go undiagnosed or misdiagnosed due to the lack of autism specialists and autism awareness. The Screening Tools for Autism Risk using Technology (START) app was created and tested in Delhi, India, to assess autism symptom severity and developmental levels. The app utilizes computerized games and activities to assess social, motor, and sensory skills. The app can be administered by non-specialists to address global disparities in access to ASD-specific care and reduce reliance on autism specialists [48]. By informing providers about autism diagnosis, utilizing technology, and expanding access to care, such programs can improve service delivery and outcomes for individuals with ASD. 

Future directions in advocacy for individuals with ASD in dermatology 

Delays in accessing medical care pose a significant challenge for children who are diagnosed with ASD as well as their caregivers. Lindly et al. identified difficulty in securing a timely doctor's visit, extended wait times in a doctor's office, and transportation issues as contributors to delayed healthcare access among those with ASD [49]. By incorporating telehealth visits, which offer patients communication and access to their healthcare provider through an electronic platform, a dermatology clinic observed a 36% increase in scheduling capacity for their patients [50]. Bypassing in-patient visits through telehealth services thus helps address the systemic issues that complicate the treatment of ASD patients by increasing appointment availability, reducing wait times, and expanding services to otherwise underserved areas. Individuals with autism are also particularly receptive to technology and benefit from augmentative and alternative communication devices, including computers, tablets, or mobile applications that offer features such as text-to-voice [51]. By incorporating these advances in medical practice, patients with autism can enhance communication skills with their health providers and properly advocate for their needs within a dermatology setting. Artificial intelligence (AI) can be adopted to improve care for patients with ASD as well. Wu et al. demonstrated that common ASD comorbidities, including psoriasis and AD, were diagnosed by AI with a 95.80% accuracy when compared to dermatologist-confirmed diagnoses [52]. High rates of diagnostic concordance between AI and dermatologists for these comorbidities underscore the benefits of incorporating AI into dermatological practice for patients with ASD, bridging the gap in accessing care. 

The increasing prevalence of ASD and its association with cutaneous pathologies necessitate the expansion of autism research within the scope of dermatology. Current research could be further improved through the recruitment of more patients with ASD and through the coordination of clinical trials including this patient population. To accomplish these feats, integration of autism-informed care into medical education and training is critical since physicians frequently cite their lack of expertise in behavioral management as a barrier to the care of ASD patients [53]. At the forefront of advancing autism-centered care and grant-funded research is the National Center for Autism Spectrum Disorders (NCASD), which draws on the expertise of an interdisciplinary team of psychiatrists, psychologists, speech and language pathologists, and interns to form evidence-based guidelines for the evaluation and treatment of ASD [54]. Medical training sessions can incorporate this collaborative approach through online platforms to reach a larger audience. As demonstrated by Becevic et al., health professionals reported greater self-efficacy in the assessment and treatment of common comorbidities and the ability to provide care for those with ASD following telementoring sessions from an ASD expert panel comprised of a behavioral and developmental physician, a psychiatrist, medical specialists, a clinical psychologist, a community resource specialist, and a parent advocate [55]. Consequently, through a multidisciplinary approach for patients with autism involving primary care, therapists, public health, and specialists, specifically dermatologists, evidence-based guidelines can be shared and tailored to patients, thus promoting equitable care for individuals with ASD. 

## Conclusions

Advancing dermatologic care for individuals with ASD requires a transformation in both clinical practice and healthcare delivery systems to account for the distinct challenges experienced by this population. Sensory processing differences, such as hypersensitivity to light, sound, and touch, can render standard dermatologic examinations and procedures overwhelming or intolerable. Communication differences, including limited verbal expression or difficulty interpreting social cues, may impede accurate history-taking, delay diagnoses, and compromise adherence to treatment regimens. Compounding these challenges is a disproportionate burden of dermatologic conditions, including AD, acne excoriée, and self-injurious skin behaviors, that often go undertreated due to inadequate clinician training and inaccessible care environments. Improving outcomes requires not only physical modifications to clinical spaces, such as reducing ambient noise, dimming lights, and offering visual schedules, but also meaningful shifts in clinician education and health policy. High-priority interventions include integrating ASD-specific competencies into dermatology residency curricula and continuing medical education, standardizing the use of sensory-adaptive strategies across dermatology clinics, and expanding public and private insurance mandates to cover preventive and non-urgent dermatologic services for neurodiverse patients. Collaboration among dermatologists, developmental specialists, and mental health providers supports the development and delivery of comprehensive care strategies tailored to the clinical, sensory, and behavioral needs of individuals with ASD. A care model informed by current scientific evidence and principles of neurodiversity has the potential to enhance diagnostic precision, optimize treatment adherence, and improve overall patient experience while also meeting the ethical responsibility to provide dermatologic care for individuals with ASD that is equitable, personalized, and inclusive. 
